# Non‐Invasive Follicular Thyroid Neoplasm With Papillary‐Like Nuclear Features (NIFTPs)

**DOI:** 10.1002/kjm2.70051

**Published:** 2025-05-31

**Authors:** Howard Her‐Juing Wu, Tieying Hou

**Affiliations:** ^1^ Department of Pathology and Laboratory Medicine Indiana University School of Medicine Indianapolis Indiana USA

**Keywords:** follicular variant of thyroid carcinoma, NIFTP, papillary thyroid carcinoma, thyroid

## Abstract

Over the past few years, follicular‐patterned thyroid nodules with nuclear features of papillary thyroid carcinoma (PTC) have been further subclassified based on molecular profiling, histologic features, and clinical behavior. Non‐invasive follicular thyroid neoplasm with papillary‐like nuclear features (NIFTPs) was established as a distinct entity in the 2017 4th edition of the *WHO Classification of Tumors of Endocrine Organs* due to its indolent clinical behavior and excellent prognosis. These tumors are characterized by a well‐defined border, an exclusive follicular growth pattern, and PTC nuclear features. Molecularly, NIFTPs typically harbor *RAS* gene mutations, *PAX8‐PPARG* rearrangements, or *THADA* gene fusions. In the 2022 5th edition of the *WHO Classification*, subcentimeter NIFTPs and oncocytic NIFTPs were introduced as new subtypes. In this review, we summarize the epidemiology, ultrasound features, histology/cytology, and molecular characteristics of NIFTP. As research continues to refine diagnostic criteria and management approaches, recognizing the distinct molecular and clinical features of NIFTP is essential for optimizing patient care and ensuring appropriate surgical management.

## 1 Introduction

Non‐invasive follicular thyroid neoplasm with papillary‐like nuclear features (NIFTP) is a recently defined thyroid tumor with very low risk, previously known as encapsulated non‐invasive follicular variant papillary thyroid carcinoma (EFVPTC) [1, 2]. This diagnosis was introduced as a new entity in the 2017 4th edition of the World Health Organization (WHO) *Tumours of Endocrine Organs*. In the 2022 WHO classification, NIFTP was grouped among three low‐risk follicular cell‐derived neoplasms [3, 4].

The term follicular variant papillary thyroid carcinoma (FVPTC) was introduced by Lindsay in 1960 [5]. He described these tumors with a follicular growth pattern and nuclear features similar to papillary thyroid carcinoma (PTC). In 1977, Chen and Rosai [6] analyzed six cases and concluded that FVPTC behaved biologically like classic PTC, exhibiting nodal metastasis rather than the hematogenous spread typical of follicular carcinoma. Since then, the diagnosis of PTC has been based primarily on nuclear features, irrespective of architecture, encapsulation, or invasion.

FVPTC, including the encapsulated noninvasive subtype, has become one of the most common subtypes of PTC [7, 8, 9]. FVPTC can be subclassified into encapsulated (EFVPTC) and infiltrative tumors (IFVPTC). Several studies have shown that EFVPTC and IFVPTC are biologically, cytologically, and molecularly distinct. The IFVPTC are more likely associated with the *BRAF V600E* mutation and lymph node metastasis, while EFVPTC often harbors *RAS* family mutations and remains confined to the thyroid [10, 11, 12]. There is also a notable difference in the cytologic diagnosis between noninvasive EFVPTC and IFVPTC. In Ibrahim and Wu's study, 75% of IFVPTC cases were classified by fine needle aspiration (FNA) as suspicious for or diagnostic of PTC, compared to only 4% of EFVPTC cases with the same diagnosis (*p* < 0.05) [13]. EFPTC cases often exhibit subtle and patchy nuclear changes, posing a significant challenge for pathologists in distinguishing between follicular adenoma and FVPTC. The overdiagnosis of FVPTC has resulted in overtreatment, including total thyroidectomy and unnecessary radioactive iodine therapy. To address this issue, a 2016 working group of 24 experienced thyroid pathologists, led by Nikiforov, established consensus diagnostic criteria for EFVPTC. Among 109 patients with noninvasive EFVPTC treated with lobectomy alone, without radioactive iodine ablation, all remained alive and disease‐free at a follow‐up of 10–26 years. In contrast, adverse events occurred in 12 of 101 (12%) cases of invasive EFVPTC. Based on these clinical outcomes, the term NIFTP was adopted [1].

## 2 Histological Criteria for NIFTP

The consensus diagnostic features of encapsulated FVPTC established by the 2016 working group outlined a comprehensive list of inclusion and exclusion criteria, categorized into major and minor diagnostic features for EFVPTC [1]. The major inclusion features for EFVPTC include encapsulation or clear demarcation, a follicular growth pattern, and nuclear features characteristic of PTC. Minor inclusion features consist of dark colloid, irregularly shaped follicles, intraluminal fibrosis, the sprinkling sign, follicles cleft from the stroma, and multinucleated giant cells within follicles. Exclusion features include true papillae > 1%, psammoma bodies, an infiltrative border, tumor necrosis, high mitotic activity, and morphologic characteristics of other variants of PTC. The nuclear features of PTC were further categorized into three groups: (1) size and shape (nuclear enlargement, overlapping, crowding, elongation), (2) nuclear membrane irregularities (irregular contours, grooves, pseudoinclusions), and (3) chromatin characteristics (clearing with margination, glassy nuclei). A three‐point scoring system was used to assess the degree of nuclear characteristics typical of PTC. The most accurate classification was achieved when a score of 2 or 3 was required for the diagnosis of NIFTP. The 2016 initial diagnostic criteria for NIFTP include: (1) Encapsulation or clear demarcation, (2) a follicular growth pattern with < 1% papillae, no psammoma bodies, and < 30% solid/trabecular/insular growth pattern, (3) a nuclear score of 2–3, (4) no vascular or capsular invasion, (5) no tumor necrosis, and (6) no high mitotic activity.

Following the publication of the NIFTP diagnostic criteria, several large retrospective studies reported on the clinical outcomes of patients. Most studies showed no tumor‐related adverse events after a mean follow‐up ranging from 5.8 to 11.2 years [9, 14, 15]. However, two studies indicated that adverse outcomes, including nodal and distant metastasis, occurred in 6% of patients with nodules meeting the diagnostic criteria for NIFTP [16, 17]. Some of these cases with adverse outcome showed less than 1% of papillae, revealed the *BRAF V600E* mutation and had synchronous papillary microcarcinoma [17]. To avoid misdiagnosing clinically aggressive tumors as NIFTP, Nikiforov et al. [2] proposed to replace the criterion of “less than 1% papillae” with “no well‐formed papillae.” It was also suggested that for tumors with pronounced nuclear features of PTC (nuclear score 3), the entire tumor should be submitted for microscopic examination to rule out the presence of true papillary structures. Additionally, if ancillary studies reveal the presence of *BRAF V600E, BRAF V600E*‐like mutations (e.g., *RET/PTC* fusions), or high‐risk mutations (e.g., *TERT, TP53*), this should prompt a thorough search for invasive features and true papillary structures [2] (Table 1).

**TABLE 1:** Revised diagnostic criteria for NIFPT [2].

Primary	Secondary
Encapsulation or clear demarcation Follicular growth pattern with – No well‐form papillae – No psammoma bodies – < 30% solid/trabecular/insular growth pattern Nuclear score 2–3 No vascular or capsular invasion No tumor necrosis or high mitotic activity	Lack of BRAF V600E mutation detected by molecular assays or immunohistochemistry Lack of BRAF V600E‐like mutation or other high‐risk mutations (TERT, TP53)

## 3 Epidemiology

Nikiforov et al. documented an NIFTP rate of 18.6%, which was soon followed by several other studies from the United States and European countries, suggesting similar rates ranging from 15% to 28% [1, 9, 14]. The adoption of this terminology has reduced the frequency of a histologic diagnosis of thyroid cancer. A recent meta‐analysis included 50 retrospective studies with 100,780 PTCs and 3990 NIFTP from 92 institutions worldwide [18]. The overall incidence of NIFTP was 6.0%. The incidence of NIFTP was similar in North America and Europe (9.3% vs. 9.6%), but significantly lower in Asia (2.1%). The introduction of NIFTP may prevent over 30,000 patients worldwide annually from being diagnosed with thyroid malignancies [18].

## 4 Cytology

**FIGURE 1:**
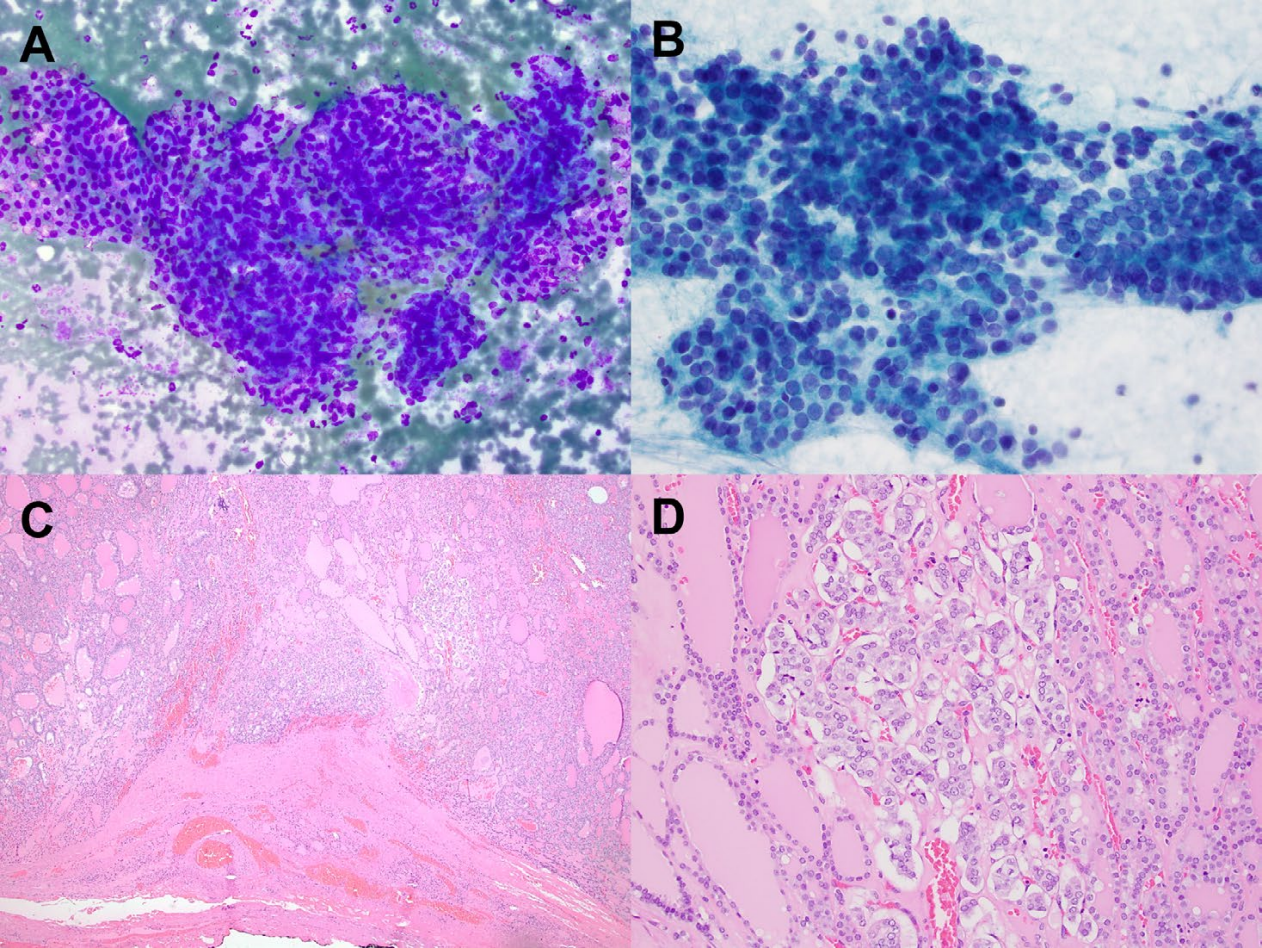
(A and B) Fine needle aspiration (FNA) from a 3.4 cm thyroid mass revealed cellular smears with atypical follicular cells demonstrating nuclear enlargement, crowding, and chromatin clearing. The FNA diagnosis was atypia of undetermined significance (AUS). ThyroSeq identified an *HRAS* mutation, indicating an intermediate to high risk of malignancy (70%). (C and D) Surgical resection showed a partially encapsulated mass with a predominantly follicular pattern and patchy papillary‐like nuclear features.

**FIGURE 2:**
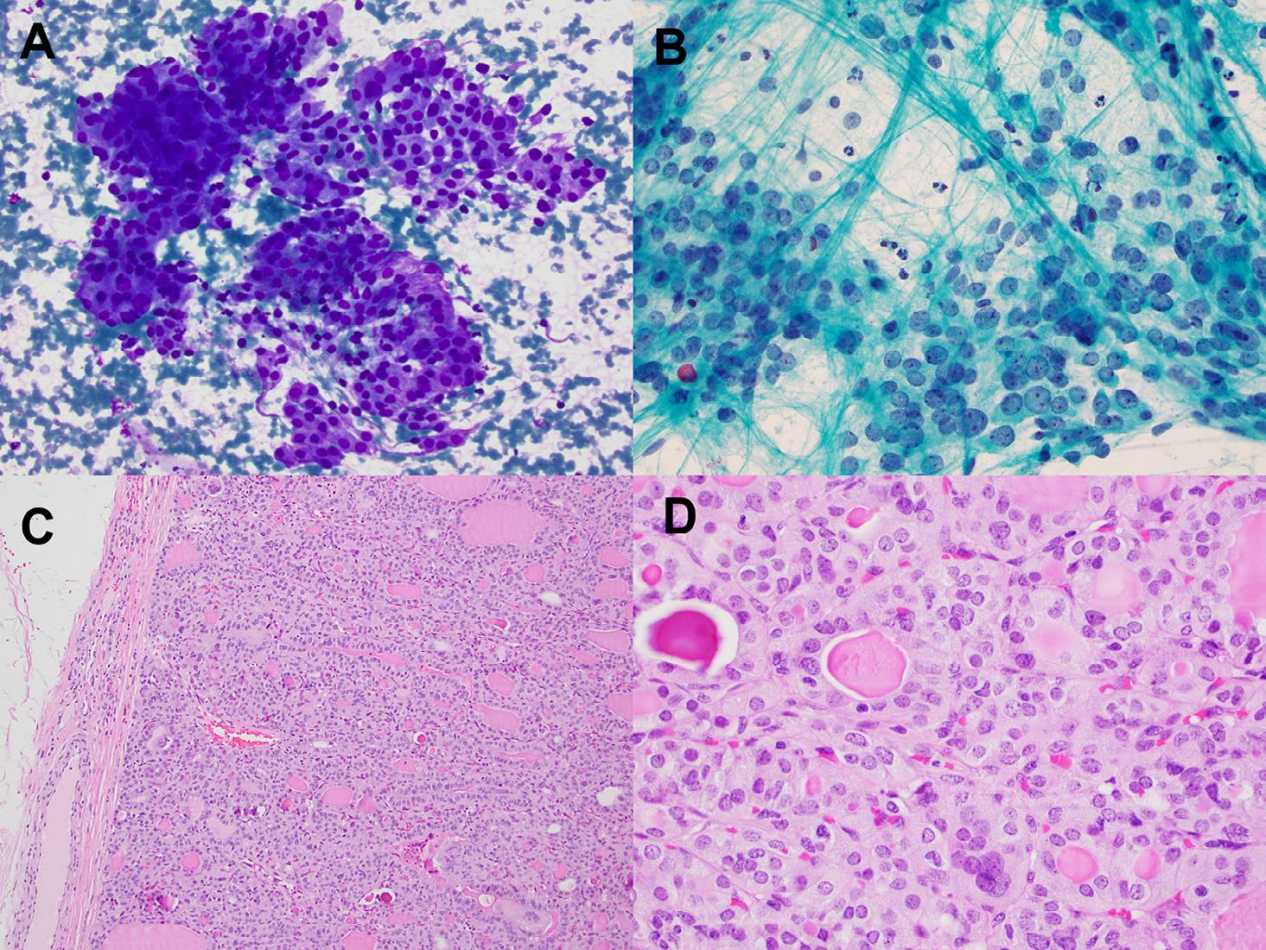
(A and B) FNA of a 3.9 cm thyroid mass in an 11‐year‐old female demonstrated hypercellular smears with numerous clusters of atypical follicular cells. These cells exhibited nuclear enlargement, open chromatin, small nucleoli, and occasional nuclear grooves, along with a moderate amount of granular cytoplasm. The FNA diagnosis was suspicious for papillary thyroid carcinoma. ThyroSeq detected an *NRAS* mutation with gene expression alterations, corresponding to a 70%–80% risk of malignancy. (C and D) Surgical resection revealed a well‐defined mass with a thin capsule. Patchy nuclear atypia, including irregular nuclear membranes, crowding, and chromatin clearing, was observed.

In approximately 75%–80% of cases, the preoperative FNA cytologic diagnosis of NIFTP falls into indeterminate categories within the Bethesda System for Reporting Thyroid Cytopathology. Of these, 50%–75% are diagnosed as atypia of undetermined significance (AUS), 25%–30% as follicular neoplasm (FN), and a minority as suspicious for malignancy [19, 20, 21, 22, 23]. The reclassification of the non‐invasive follicular variant of papillary carcinoma into NIFTP most significantly affected the risk of malignancy in three intermediate categories of the Bethesda System for Reporting Thyroid Cytopathology: AUS, FN, and suspicious for malignancy. In a multi‐institutional study, the AUS category showed a decreased risk of malignancy, ranging from 5.2% to 13.6%; the FN category had a decrease of 9.9% to 15.1%; and the suspicious for malignancy category saw a reduction in risk of 17.6% to 23.4% [19]. The smears from FNA biopsies of NIFTP typically show low to moderate cellularity. The cellular changes include a predominance of microfollicles or at least a focal follicular growth pattern, often accompanied by a mixture of syncytial sheets and microfollicles. Both NIFTP and invasive PTC exhibit nuclear clearing. However, compared to invasive PTC, the nuclei of NIFTP are smaller, less elongated, and more rounded. NIFTP nuclei are also less crowded, with more focal and delicate grooves compared to those of PTC. Papillae, psammoma bodies, and multiple nuclear pseudoinclusions are typically absent in NIFTP cases [13, 20, 21, 22, 23, 24] (Figures 1 and 2).

## 5 Ultrasonographic (US) Studies

The US features of most NIFTP are not highly suspicious. The ultrasound findings for NIFTP typically demonstrated a circumscribed oval/round solid (hypo‐ or iso‐echoic) nodule with a hypoechoic rim, absence of microcalcifications, and the Doppler was most hypervascular [23, 25]. The Thyroid Imaging Reporting and Data System (TI‐RADS) uses ultrasound features to calculate the score. For TI‐RADS score, the majority of NIFTP were assigned as TR3 (mildly suspicious) and TR4 (moderately suspicious) [26].

## 6 Molecular Studies

There are no specific molecular alterations unique to NIFTP; however, it shares some molecular mutations with other follicular‐patterned thyroid neoplasms, including follicular adenoma, follicular thyroid carcinoma, and follicular variant of PTC. The most common mutations found in this group of tumors are point mutations in the *RAS* gene family and *PAX8:PPARG* rearrangements. Most NIFTP cases carry either *RAS* point mutations (up to 60%), *PAX8:PPARG* rearrangement (up to 30%) or T*HADA* gene fusions (up to 30%) and rare occurrences of *BRAF K601E*, *EIF1AX, EZH1, DICER1, PTEN*, and *TSHR* mutations (< 10%). The characteristic mutations for conventional PTC, such as *BRAF V600E* and *RET* fusions, are almost always absent in the NIFTPs [27, 28, 29, 30].

In addition to molecular genetic testing, several studies have explored the utilization of epigenetic analysis in thyroid neoplasms, including microRNA (miRNA) and mRNA. Among the miRNA, downregulation of miR‐7‐5p discriminates NIFTP from hyperplasia, while upregulation of miR‐222‐3p distinguishes FVPTC from NIFTP. Classic PTC is associated with high levels of miR‐146b‐5p [31]. High expression of three miRNAs (miR‐136, miR‐21, and miR‐127) has been demonstrated in differentiated thyroid cancer, while low expression levels were found in benign tumors or NIFTPs [32].

Lee et al. [33] performed mRNA expression profiling for 74 fresh frozen thyroid tissue samples, including NIFTP as well as benign and malignant follicular‐cell‐derived tumors. Their study suggests that OCLN, ZNF423, LYG1, and AQP5 mRNA markers could serve as useful molecular markers for identifying NIFTP among other thyroid tumors. In NIFTP, two of the four mRNAs investigated (OCLN and ZNF423) showed upregulation, while the other two (LYG1 and AQP5) displayed downregulation.

## 7 Sub‐Centimeter NIFTPs

**FIGURE 3:**
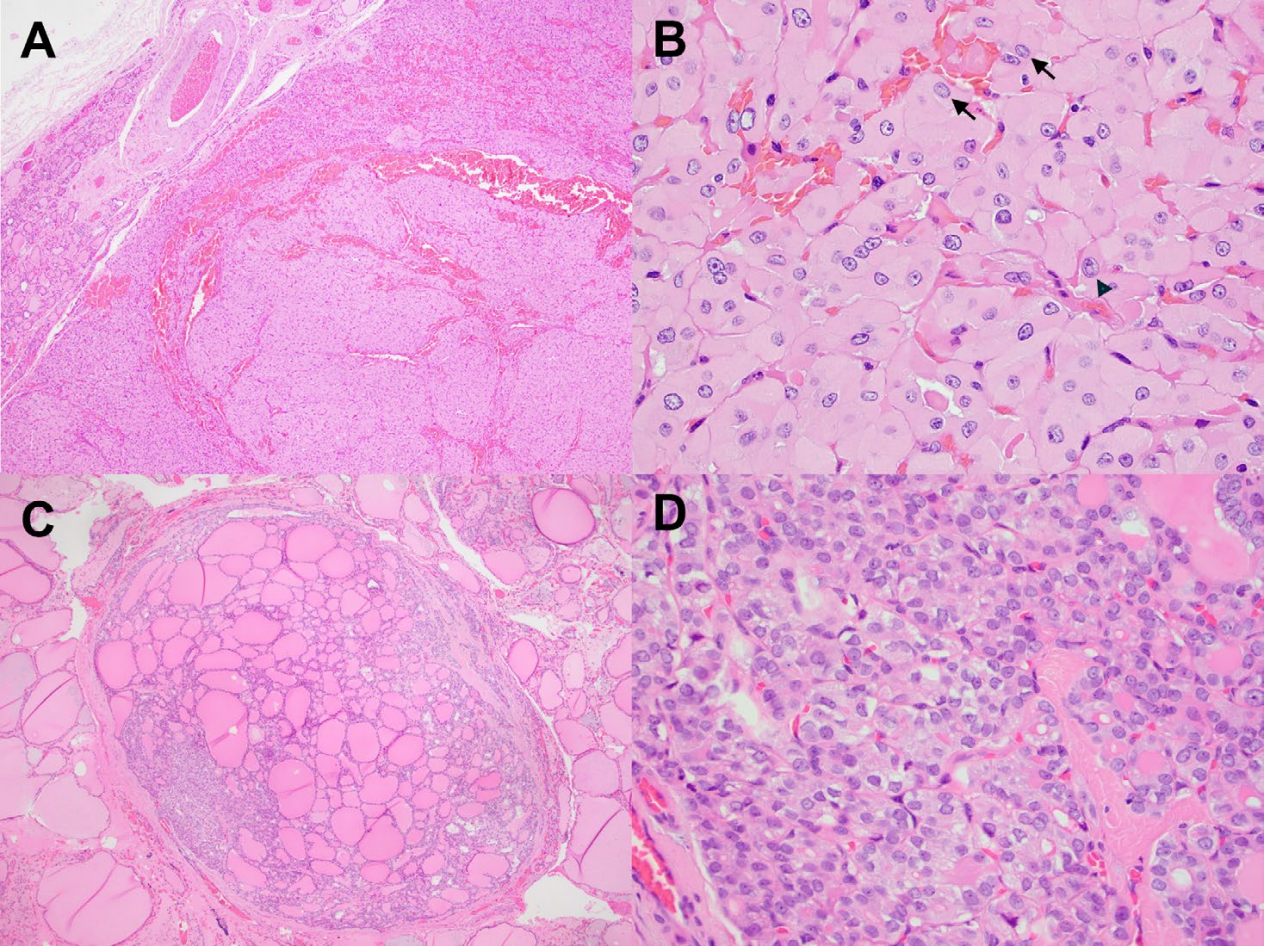
(A and B) An example of oncocytic NIFTP (1.0 cm) showed a well‐defined nodule comprising exclusively oncocytic follicular cells on H&E sections. Tumor cells displayed irregular nuclear membranes, open chromatin, nuclear grooves (arrow), and occasional intranuclear pseudoinclusions (arrowhead). (C and D) An example of a subcentimeter NIFTP (0.4 cm) showed a small, well‐circumscribed nodule with papillary‐like nuclear features arranged in a follicular pattern.

The consensus study group that established the criteria for NIFTP did not include sub‐centimeter tumors in their cohort [1]. Many sub‐centimeter tumors that meet the criteria of NIFTPs are still diagnosed as papillary thyroid microcarcinoma, and some of these patients received unnecessary total thyroidectomy. A multi‐institutional study [34] included 52 patients with unifocal, micropapillary thyroid carcinoma, noninvasive encapsulated follicular variant from five tertiary hospitals. All patients had at least 1 year of clinical follow‐up without post‐operative radio‐iodine treatment. Among them, 23 patients (44%) underwent lobectomy alone, while the remaining 29 patients (56%) received total thyroidectomy. No recurrence was observed in the entire cohort. In another study, Shafique et al. [35] also demonstrated that sub‐centimeter papillary carcinoma can fulfill all the criteria of NIFTP while also exhibiting indolent behavior. Sub‐centimeter micropapillary thyroid carcinomas that meet the stringent inclusion criteria for NIFTP should be classified accordingly to prevent overtreatment [34, 35]. (Figure 3).

## 8 Large (> 4 cm) NIFTPs

Before the introduction of the NIFTP terminology, large NIFTPs measuring at least 4 cm were staged as pT3a, and post‐operative radioactive iodine treatment was often considered a management option. An international collaborative study examined 79 patients with NIFTP tumors measuring at least 4 cm. Among these, 37 patients did not receive radioactive iodine treatment, yet all patients remained disease‐free with a median follow‐up of 6.7 years. When the stringent criteria are met, large tumors (> 4 cm) should be diagnosed as NIFTP. For this group of large tumors, conservative surgical treatment is sufficient, and additional postoperative radioactive iodine treatment is not required [36, 37].

## 9 NIFTPs in Pediatric Patients

Literature on NIFTPs in pediatric patients is limited. These tumors account for approximately 14% of all follicular‐patterned thyroid nodules with nuclear features of PTC, and 1.9% of resected thyroid nodules in pediatric patients [38, 39]. In retrospective studies, pediatric NIFTPs were historically diagnosed as EFVPTC, with most patients undergoing total thyroidectomy [38, 40]. These tumors were typically early‐stage (T1 or T2) and did not have lymph node metastasis [40, 41]. Similar to adults, all pediatric patients with NIFTPs remained disease‐free during clinical follow‐up [38, 39, 40, 41]. This suggests that pediatric NIFTPs should be managed with conservative lobectomy as in adult patients.

## 10 Oncocytic NIFTPs

Oncocytic NIFTPs represent a subtype of NIFTP introduced in the 2022 5th edition of *WHO Classification of Tumours of Endocrine Organs* [4]. Similar to other oncocytic FNs, they are defined as NIFTPs in which more than 75% of tumor cells exhibit eosinophilic granular cytoplasm (Figure 3). Molecularly, oncocytic NIFTPs share alterations with both follicular and oncocytic neoplasms. Xu et al. [42] conducted molecular profiling on 15 cases of non‐invasive EFVPTC with oncocytic features, revealing molecular characteristics that overlap with both PTC with follicular pattern and oncocytic neoplasms. *RAS* mutations were the most common driver mutation, detected in 33% of cases. Nonsilent mitochondrial DNA mutations were identified in 67% of tumors, a feature shared with oncocytic carcinoma. Additionally, 53% of cases demonstrated multiple chromosomal gains and losses. However, none of the tumors showed global uniparental disomy, a characteristic of oncocytic carcinoma. In this series, none of the 33 patients with more than 5 years of follow‐up experienced recurrence [42].

## 11 Conclusions

Since the introduction of NIFTP as a distinct entity in the WHO tumor classification of endocrine organs in 2017, with refined diagnostic criteria established in 2022, extensive research has provided valuable insights into its pathological, molecular, radiological, and clinical characteristics. These studies consistently support the concept of NIFTP as a clinically indolent neoplasm with an extremely low malignant potential, warranting conservative management through lobectomy. Continued investigation, particularly into pediatric and oncocytic variants of NIFTP, will further enhance our understanding of this tumor and guide the refinement of surgical management strategies.

## Conflicts of Interest

The authors declare no conflicts of interest.

## Data Availability

The authors have nothing to report.
